# The effect of rTMS in the management of pain associated with CRPS

**DOI:** 10.1515/tnsci-2020-0120

**Published:** 2020-09-28

**Authors:** Min Cheol Chang, Sang Gyu Kwak, Donghwi Park

**Affiliations:** Department of Rehabilitation Medicine, College of Medicine, Yeungnam University, Daegu, Republic of Korea; Department of Medical Statistics, College of Medicine, Catholic University of Daegu, Daegu, Republic of Korea; Department of Physical Medicine and Rehabilitation, Ulsan University Hospital, University of Ulsan College of Medicine, 877, Bangeojinsunhwando-ro, Dong-gu, 44033, Ulsan, Republic of Korea

**Keywords:** complex regional pain syndrome, repetitive regional pain syndrome, pain, meta-analysis

## Abstract

**Background:**

Therapeutic management of pain in patients with complex regional pain syndrome (CRPS) is challenging. Repetitive transcranial magnetic stimulation (rTMS) has analgesic effects on several types of pain. However, its effect on CRPS has not been elucidated clearly. Therefore, we conducted a meta-analysis of the available clinical studies on rTMS treatment in patients with CRPS.

**Materials and methods:**

A comprehensive literature search was conducted using the PubMed, EMBASE, Cochrane Library, and SCOPUS databases. We included studies published up to February 09, 2020, that fulfilled our inclusion and exclusion criteria. Data regarding measurement of pain using the visual analog scale before and after rTMS treatment were collected to perform the meta-analysis. The meta-analysis was performed using Comprehensive Meta-analysis Version 2.

**Results:**

A total of three studies (one randomized controlled trial and two prospective observational studies) involving 41 patients were included in this meta-analysis. No significant reduction in pain was observed immediately after one rTMS treatment session or immediately after the entire schedule of rTMS treatment sessions (5 or 10 sessions; *P* > 0.05). However, pain significantly reduced 1 week after the entire schedule of rTMS sessions (*P* < 0.001).

**Conclusion:**

rTMS appears to have a functional analgesic effect in patients with CRPS.

## 1 Introduction

Complex regional pain syndrome (CRPS) is a chronic, painful condition that can affect any part of the body, but it commonly affects the limbs [1,2,3,4,5,6] CRPS involves vasomotor changes such as changes in color and temperature of the skin, edema, increased sensitivity to touch, and a limited range of movement [1,2]. CRPS occurs due to any damage or injury to the central or peripheral nervous system and can also develop after direct trauma to the limbs [1,2]. CRPS is divided into two types – types I and II. CRPS type II is associated with a confirmed peripheral nerve injury, while CRPS type I is not associated with an obvious peripheral nerve injury [1,2].

The pain associated with CRPS is often severe and unresponsive to various treatment modalities, procedures, or oral administration of pharmacotherapeutic agents. Therefore, several patients with CRPS have severe, refractory pain that affects their quality of life and might result in unemployment [7,8]. In addition, the long-lasting, severe pain can result in psychological disorders such as depression and anxiety [9]. Therefore, controlling CRPS-induced pain is a challenge in clinical practice.

Repetitive transcranial magnetic stimulation (rTMS) is a safe, noninvasive, and effective therapeutic intervention, in which an electromagnetic coil is placed on the scalp to create a magnetic field [10,11,12,13,14]. The resulting magnetic stimulation changes the cortical excitability of the stimulation area and the distant areas transsynaptically. High-frequency (≥5 Hz) stimulation increases cortical excitability, while low-frequency (≤3 Hz) stimulation decreases the excitability [12]. Various types of chronic pain such as neuropathic pain, musculoskeletal pain, and fibromyalgia have been effectively controlled using rTMS [10,11,14,15]. Moreover, some studies have reported a positive pain-controlling effect of rTMS in patients with CRPS [16,17,18,19,20,21]. However, since the aforementioned studies on rTMS are limited by their small sample size, the effect of rTMS on CRPS has not been clearly elucidated. However, previous review articles have reported that rTMS might be useful to alleviate pain in patients with CRPS [19,20,21].

To evaluate the effectiveness of rTMS in controlling pain associated with CRPS, we conducted a meta-analysis of all available clinical studies involving rTMS treatment in patients with CRPS.

## 2 Methods

### 2.1 Search strategy

This meta-analysis was conducted in accordance with the preferred reporting items for systematic review and meta-analysis guidelines. The following databases were systematically searched for relevant studies published until February 09, 2020: PubMed, SCOPUS, EMBASE, and Cochrane Library. The key words used for the search were “(CRPS AND rTMS) OR (reflex sympathetic dystrophy AND rTMS).”

### 2.2 Eligibility criteria

Articles were included in this meta-analysis if they met the following criteria: (1) patients’ pain was induced by CRPS, (2) rTMS was used to manage the pain, (3) the pain levels were evaluated before and after rTMS treatment, and (4) studies involved human subjects. We included all studies published in English language, without any limitations on the study design such as randomized controlled trials (RCTs). Review articles, letters, or case reports and studies that reported inadequate data/results were excluded.

### 2.3 Study selection and data extraction

After the exclusion of duplicate publications, two independent reviewers (Donghwi Park [DP] and Min Cheol Chang [MCC]) evaluated the inclusion eligibility of potential articles. Articles were screened for eligibility based on a review of the title and abstract, and disagreements were resolved by consensus. After the primary screening, the full texts of eligible articles were reviewed independently by two reviewers (DP and MCC). Subsequently, data on the first author, year of publication, sample size, demographic data, methods of rTMS, outcome measures (visual analog scale [VAS] scores), and major adverse effects were independently extracted from each eligible study (Table 1).

**Table 1:** Characteristics of the included studies

Publication year	Design	Included patients (*n*)	Disease duration (months)	rTMS mode	Outcome assessment time	Major adverse effect (N)
Gaertner et al. (2018)	Observational study	19	64.8 ± 45.6	10 Hz, 80% RMT (10 Hz for 10 s with an intertrain interval of 30 s) a total of 2,600 pulses	Pre-rTMS, immediately after 1 session, immediately after 5 sessions, 1 week after finishing 5 sessions	0
Picarelli et al. (2010)	Randonmized controlled trial	12	80.8 ± 32.1	10 Hz, 100% RMT (10 Hz for 10 s with 60 s intertrain interval), a total of 2,500 pulses	Pre-rTMS, immediately after 1 sessions, immediately after 10 sessions, 1 week after finishing 10 sessions	1 (generalized seizure)
Pleger et al. (2004)	Observational study	10	35 (range 24–72)	10 Hz, 110% of RMT (a series of ten rTMS applications, each lasting 1.2 s in length), intertrain interval 10 s	Pre-rTMS, immediately after 1 sessions	

### 2.4 Quality assessment

The methodological quality of the studies was assessed using two different tools. The Cochrane Collaboration’s Handbook was used to determine adequate sequence generation, allocation concealment, blinding, incomplete outcome data, selective outcome reporting, and other potential sources of bias in RCTs. Bias was categorized as “low risk,” “high risk,” or “unclear risk” [22]. The Newcastle–Ottawa scale (NOS) was used for assessing the quality of prospective observational studies based on three parameters – selection of subjects, comparability of groups, and assessment of outcome. The quality of each study was graded as low (score, 0–3), moderate (score, 4–6), and high (score, 7–9) [23]. All disagreements were resolved by consensus.

## 3 Statistical analysis

The extracted data were statistically analyzed using comprehensive meta-analysis version 2 (Biostat Inc.). For each analysis, a heterogeneity test was performed using *I* ^2^ statistic, which measures the extent of inconsistency among the results. When the *P* values were <0.05, the pooled data were considered having substantial heterogeneity, and the random-effects model was used for data analysis. When the *P* values were ≥0.05, the pooled data were considered homogenous and the fixed effects model was used for data analysis. Since the VAS scores are continuous variables, we analyzed the standardized mean difference (SMD) in the change from baseline and 95% confidence interval (CI). Subgroup analyses were performed according to the evaluation time points. The *P* values <0.05 were considered statistically significant.

## 4 Results

### 4.1 Study selection

**Figure 1:**
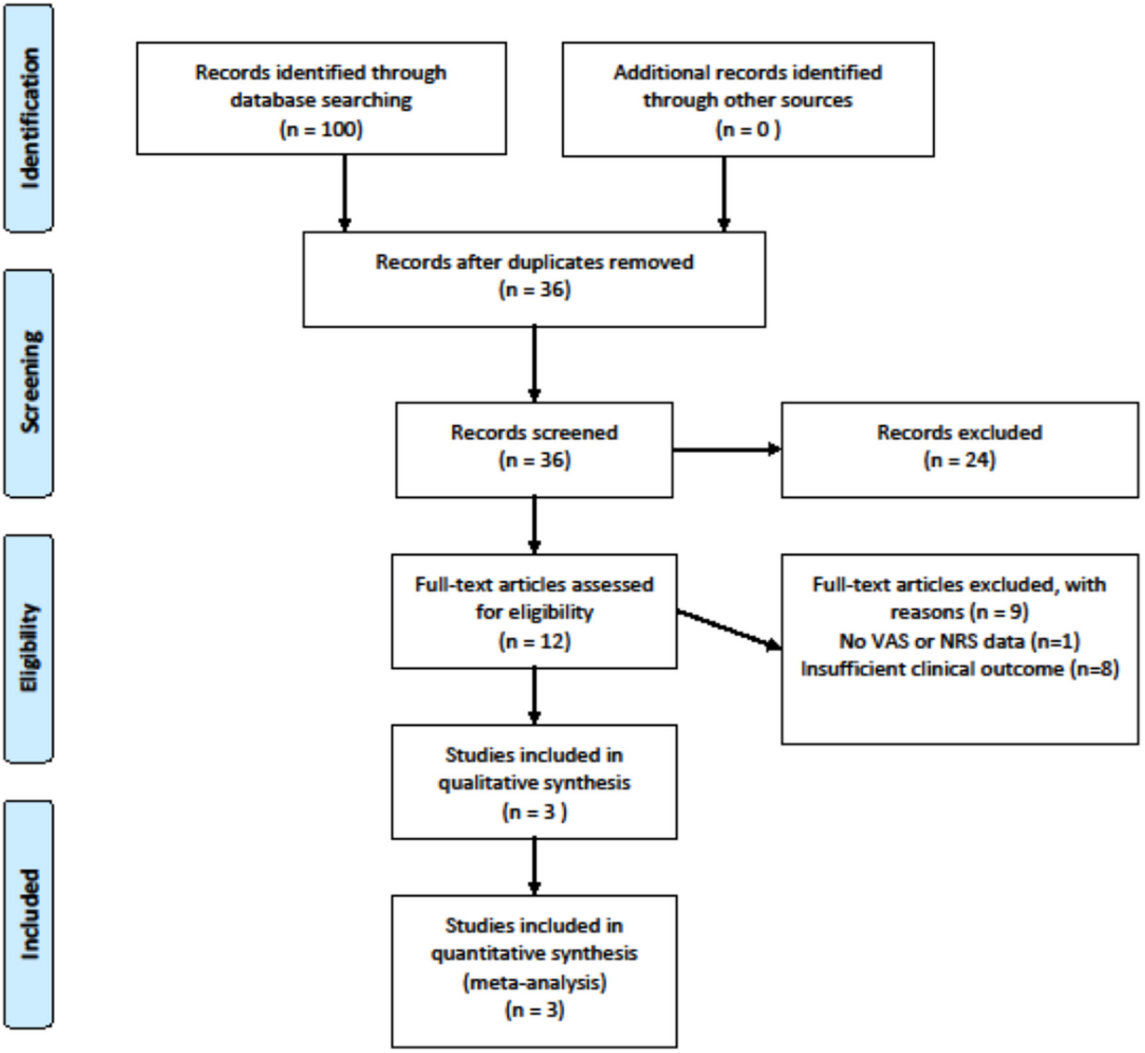
Flowchart showing the search results of the meta-analysis.

The preliminary search of all the databases provided a total of 100 potentially relevant studies (Figure 1). After the elimination of duplicate studies, 66 articles were excluded based on the review of their titles and abstracts. The remaining studies were assessed by reviewing the full text of the articles. After a systematic review, three articles were included in the final analysis [16,17,18], which included one RCT [17] and two prospective observational studies [16,18]. Of the three studies, one was an open-label trial [16], one was a parallel study [17], and one was a crossover study. [18]

The data of patients who received rTMS treatment were extracted from the RCT conducted by Picarelli et al. [17]. Picarelli et al. [17] and Pleger et al. [18] administered rTMS treatment to patients with CRPS type I, and Gaertner et al. [16] administered rTMS treatment to patients with both CRPS types I and II.

### 4.2 Study characteristics

The selected studies included 49 cases. Pleger et al. [18] administered only one rTMS treatment session and evaluated its analgesic effect in 10 patients immediately after the session. Gaertner et al. [16] evaluated the analgesic effect of one rTMS treatment session in five patients and evaluated the change in the intensity of pain immediately and 1 week after five rTMS treatment sessions in 12 patients. Picarelli et al. [17] administered 10 rTMS treatment sessions in 22 patients and measured the intensity of pain immediately after the first rTMS session and immediately after the 10 sessions. Furthermore, follow-up evaluations were performed 1 week after the 10 rTMS treatment sessions.

### 4.3 Risk of bias

The study by Picarelli et al. was an RCT [17], and the risk of bias was assessed based on the Cochrane Handbook 5.1 Assessment Tool. The risk of bias for random sequence generation, allocation concealment, blinding of participants and personnel, and blinding of the outcome assessment was unclear. A low risk of bias was observed for incomplete outcome data, selective reporting, and other biases. The other two observational studies were assessed using NOS [16,18]; Gaertner et al.’s study had a score of 12, which indicates a low risk of bias (selection of subjects: 4; comparability of groups: 4; and assessment of outcome: 4), and Pleger et al.’s study had a score of 6, which indicates a moderate risk of bias (selection of subjects: 2; comparability of groups: 1; and assessment of outcome: 3).

### 4.4 Results of the meta-analysis

**Figure 2:**
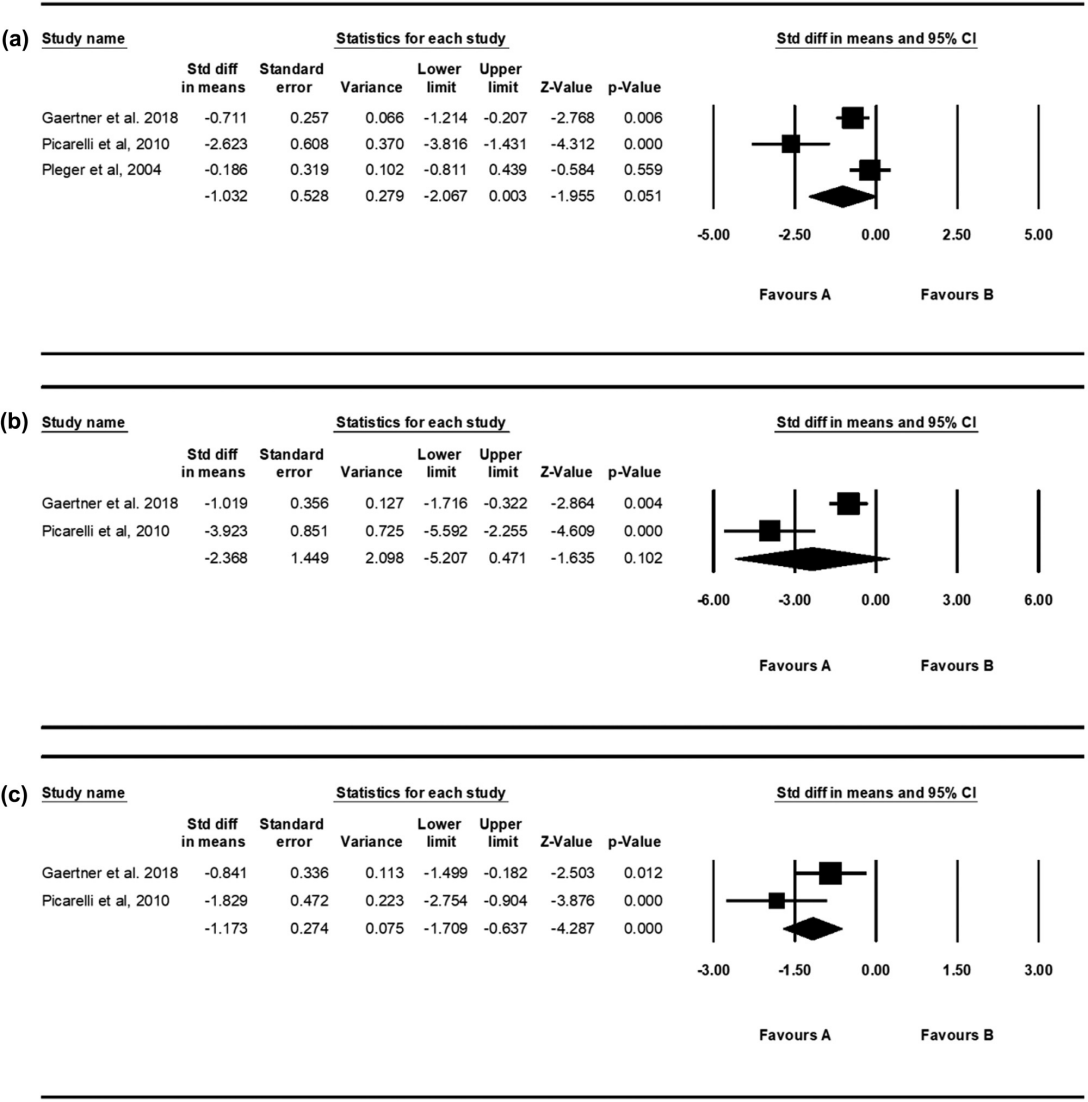
Changes in the visual analog scale scores immediately after one rTMS treatment session (a), immediately after the completion of the entire schedule of rTMS treatment sessions (5 or 10 sessions) (b), and 1 week after the completion of the entire schedule of rTMS sessions (5 or 10 sessions) (c).

We analyzed the effect of rTMS immediately after one rTMS treatment session and immediately after the entire schedule of rTMS treatment sessions (5 or 10 sessions). Additionally, the effect of rTMS was evaluated 1 week after the entire schedule of rTMS treatment sessions. Since the *P* value for heterogeneity of the assessments that were performed immediately after one rTMS treatment session and immediately after the entire schedule of rTMS treatment sessions (5 and 10 sessions) was <0.05, the random-effects model was used (immediately after one rTMS session: *P* = 0.002, *I* ^2^ = 84.114, tau^2^ = 0.677, *df* = 2; immediately after all rTMS sessions [5 and 10 sessions]: *P* = 0.002, *I* ^2^ = 89.908, tau^2^ = 3.792, *df* = 1). The *P* value for heterogeneity of the assessment 1 week after the entire schedule of rTMS treatment sessions was >0.05; hence, the fixed-effects model was used (*P* = 0.088, *I* ^2^ = 65.676, tau^2^ = 0.321, *df* = 1; Figure 2).

An analysis of the VAS scores immediately after one rTMS treatment session and immediately after the entire schedule of rTMS treatment sessions (5 and 10 sessions) revealed no significant reduction in VAS scores (one rTMS session: SMD = −1.032, 95% CI = −2.067–0.003, *P* = 0.051; entire schedule of rTMS sessions: SMD = −2.368, 95% CI = −5.207–0.471, *P* = 0.102). However, a tendency of reduction in pain was experienced by the study subjects. One week after the entire schedule of rTMS treatment sessions, the pain significantly reduced (SMD = −1.173, 95% CI = −1.709 to −0.637, *P* < 0.001).

In the study by Gu and Chang [12], 1 of the 12 patients who received rTMS treatment developed generalized seizure. In the other two studies, no major adverse effects were reported.

### 4.5 Publication bias

**Figure 3:**
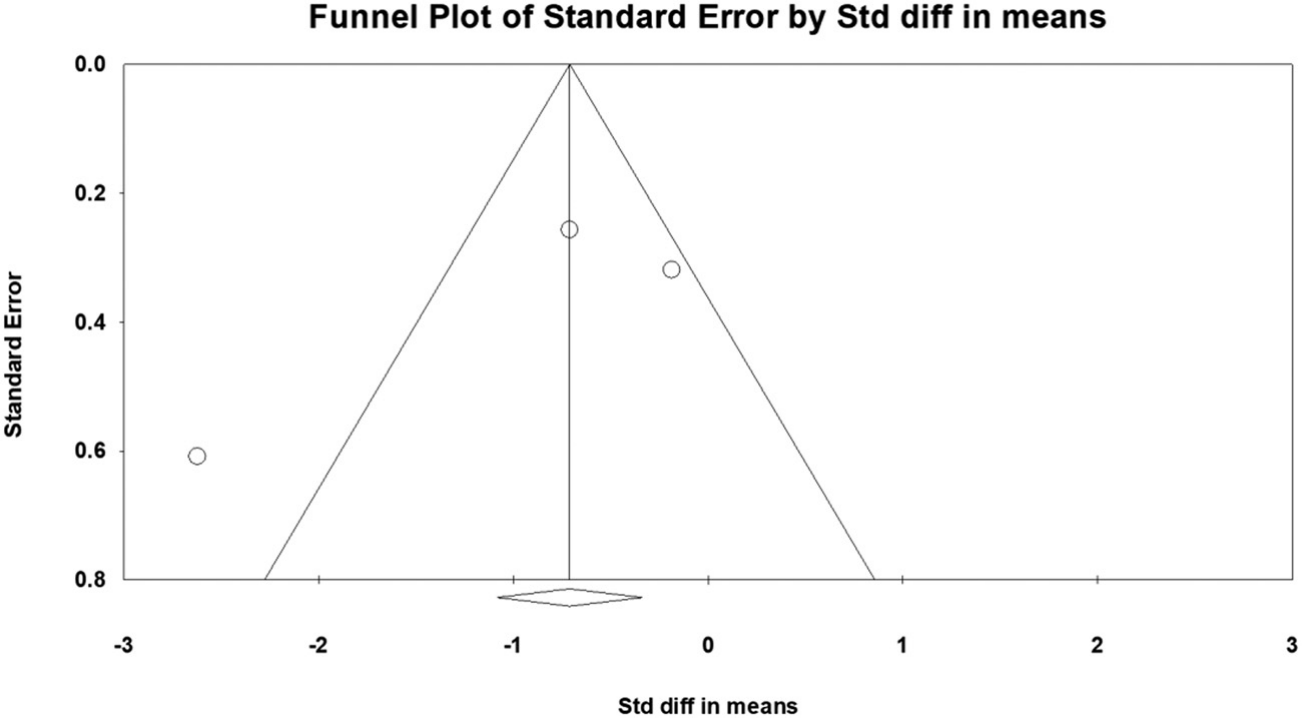
Graphic funnel plot of the included studies, depicting the change in visual analog scale scores immediately after 1 rTMS session.

A funnel plot analysis was performed for the assessment immediately after one rTMS treatment session. The graphical funnel plot, which involved the change in VAS scores reported by the studies included in this meta-analysis, appeared to be symmetrical (Figure 3). In addition, the publication bias was quantified using Egger’s test. The intercept was found at −5.945 (*P* = 0.401). Therefore, a statistically significant publication bias was unlikely to occur.

## 5 Discussion

The current meta-analysis evaluated whether rTMS can alleviate pain associated with CRPS. A total of three studies were included and the pain scores, measured using the VAS, were analyzed. No significant reduction in pain was observed immediately after one rTMS treatment session. Similarly, no significant reduction in pain was observed immediately after 5 and 10 rTMS treatment sessions. A tendency toward decrease in pain was observed after one rTMS treatment session and after the completion of the entire schedule of rTMS treatment sessions (5 or 10 sessions). However, pain associated with CRPS was significantly reduced 1 week after the entire schedule of 5 or 10 rTMS treatment sessions. The effect size of the efficacy 1 week after 5 or 10 rTMS treatment sessions was −1.173. Based on Cohen’s study [24], this effect size value can be interpreted as follows: rTMS treatment has a large positive pain-reducing effect in patients with CRPS.

Hirayama et al. evaluated the analgesic effect of rTMS according to the areas stimulated [25]. They applied rTMS on the primary motor cortex, primary sensory cortex, premotor area, and supplementary motor area and observed that the primary motor cortex is the only target that can reduce pain. All the three studies included in the current meta-analysis stimulated the hand area of the primary motor cortex. The mechanism of action of rTMS that results in the reduction of pain has not been clearly elucidated. However, some possible mechanisms have been proposed. Previous studies that used functional magnetic resonance imaging have found that rTMS results in changes in the activity of cortical and subcortical areas associated with the processing of pain and modulation, such as the orbitofrontal cortex, anterior cingulate, medial thalamus, and periaqueductal gray matter [26,27]. The studies have suggested that rTMS can modify the hyperexcited pain-related areas, which triggers the cascade of analgesic synaptic events. rTMS is believed to trigger the descending inhibitory pathways to act at the dorsal horn level, which inhibits the conduction of pain signal to the brain [28]. Additionally, patients with chronic pain have a decreased blood flow [29]. rTMS increases the cerebral blood flow to the affected areas [29,30]. Moreover, rTMS might have antinociceptive effects by influencing the endogenous opioid system in the periaqueductal gray matter [31,32].

In our study, pain was not significantly reduced immediately after one rTMS treatment session or after 5 or 10 rTMS treatment sessions. However, significant reduction in pain was observed 1 week after 5 or 10 rTMS treatment sessions. The analgesic effect of rTMS was continuous and cumulative, even after the completion of rTMS therapy. To control pain associated with CRPS, the cumulative sustained effects of rTMS, which continue after the completion of therapy sessions, seem necessary.

In the study by Picarelli et al. [17], 1 of the 12 patients experienced a seizure during rTMS treatment. Seizure is one of the most serious adverse effects of rTMS therapy [33]. Although seizure occurs at a frequency of <0.1%, factors such as preexisting neurological conditions, sleep deprivation, family history of seizures, and alcohol use can increase the risk of seizures [34].

In the current meta-analysis, we observed that rTMS therapy has the potential to control pain associated with CRPS. Since pain associated with CRPS is often refractory to various treatment methods, clinicians often find it challenging to treat patients with CRPS. Based on the results of this meta-analysis, we propose that rTMS can be a good treatment option for pain associated with CRPS. Furthermore, careful monitoring is warranted for adverse effects in patients with an increased risk of seizure.

The limitation of this study is that only three studies were included in the analysis. Therefore, subgroup analysis according to CRPS types I and II was not possible. However, to the best of our knowledge, this is the first meta-analysis evaluating the effects of rTMS on pain associated with CRPS. Further research is necessary to establish and explain the effect of rTMS in the management of pain associated with CRPS.
